# Liver Transplantation for Hepatocellular Carcinoma: How Should We Improve the Thresholds?

**DOI:** 10.3390/cancers14020419

**Published:** 2022-01-14

**Authors:** Tsuyoshi Shimamura, Ryoichi Goto, Masaaki Watanabe, Norio Kawamura, Yasutsugu Takada

**Affiliations:** 1 Division of Organ Transplantation, Hokkaido University Hospital, N-14, W-5, Kita-ku, Sapporo 060-8648, Hokkaido, Japan; 2 Department of Gastroenterological Surgery I, Hokkaido University Graduate School of Medicine, N-15, W-7, Kita-ku, Sapporo 060-8638, Hokkaido, Japan; ryoichi.goto@huhp.hokudai.ac.jp; 3 Department of Transplant Surgery, Hokkaido University Graduate School of Medicine, N-15, W-7, Kita-ku, Sapporo 060-8638, Hokkaido, Japan; masaaki@w8.dion.ne.jp (M.W.); norirv@live.jp (N.K.); 4 Department of HBP and Breast Surgery, Ehime University Graduate School of Medicine, Shitsukawa, Toon 791-0295, Ehime, Japan; takaday@m.ehime-u.ac.jp

**Keywords:** liver transplantation, hepatocellular carcinoma, selection criteria, allocation rule, down-staging

## Abstract

**Simple Summary:**

The ideal treatment for hepatocellular carcinoma (HCC) is liver transplantation (LT), which both eliminates the HCC and cures the diseased liver. Once considered an experimental treatment with dismal survival rates, LT for HCC entered a new era with the establishment of the Milan criteria over 20 years ago. However, over the last two decades, the Milan criteria, which are based on tumor morphology, have come under intense scrutiny and are now largely regarded as too restrictive, and limit the access of transplantation for many patients who would otherwise achieve good clinical outcomes. The liver transplant community has been making every effort to reach a goal of establishing more reliable selection criteria. This article addresses how the criteria have been extended, as well as the concept of pre-transplant down-staging to maximize the eligibility.

**Abstract:**

Hepatocellular carcinoma (HCC) is the third highest cause of cancer-related mortality, and liver transplantation is the ideal treatment for this disease. The Milan criteria provided the opportunity for HCC patients to undergo LT with favorable outcomes and have been the international gold standard and benchmark. With the accumulation of data, however, the Milan criteria are not regarded as too restrictive. After the implementation of the Milan criteria, many extended criteria have been proposed, which increases the limitations regarding the morphological tumor burden, and incorporates the tumor’s biological behavior using surrogate markers. The paradigm for the patient selection for LT appears to be shifting from morphologic criteria to a combination of biologic, histologic, and morphologic criteria, and to the establishment of a model for predicting post-transplant recurrence and outcomes. This review article aims to characterize the various patient selection criteria for LT, with reference to several surrogate markers for the biological behavior of HCC (e.g., AFP, PIVKA-II, NLR, 18F-FDG PET/CT, liquid biopsy), and the response to locoregional therapy. Furthermore, the allocation rules in each country and the present evidence on the role of down-staging large tumors are addressed.

## 1. Introduction

Liver transplantation for hepatocellular carcinoma (HCC) is an ideal treatment strategy not only to eliminate the tumor lesions but to replace a damaged liver that could become an origin of HCC in the future. Based on this concept, many liver transplantation procedures were performed for HCC patients in the 1980s, regardless of tumor burden, and without any criteria for eligibility. Unexpectedly, the outcomes were dismal due to a high recurrence rate after transplantation. However, based on those experiences, it was found that a good outcome can be obtained if the tumor meets certain criteria concerning the diameter and number of lesions [1,2]. As an extension of these findings, Mazzaferro reported the Milan criteria—based on a prospective cohort study in 1996 in which HCC patients with a single tumor of <5 cm in diameter or up to 3 tumors of <3 cm in diameter, with no extrahepatic involvement and no evidence of gross vascular invasion—were able to achieve a 4-year survival rate of 75% [3]. With similar results reported for many subsequent external validations, the Milan criteria heralded a new era and have been the international standard criteria for liver transplantation for HCC for over 20 years, as well as a benchmark for other criteria. However, over the last two decades, the Milan criteria, according to the tumor morphology, has come under intense scrutiny and are now largely regarded as too restrictive, thus limiting access to transplantation for many patients who would otherwise achieve good clinical outcomes. The liver transplant community has been making every effort to reach an agreement over the most reliable selection criteria.

## 2. Extended Criteria Based on Tumor Morphology (Diameter and Number of HCC Lesions)

Based on the finding that many cases exceeding the Milan criteria showed a good prognosis, attempts at expanding the criteria were made by increasing the diameter and number of HCC lesions. Yao et al., at UCSF (University of California, San Francisco) reported their new criteria in 2001. They analyzed the outcome of 70 consecutive liver transplant patients with HCC over a 12-year period and established the UCSF criteria. The HCC patients who met these criteria had survival rates of 90.0% and 75.2% at 1 and 5 years after transplantation, respectively, which was equivalent to the rates for the Milan criteria [4]. Although their first report was based on pathological data of the explanted liver, the UCSF criteria were prospectively validated based on pre-transplant imaging data, and they reported that the UCSF criteria could offer a potential benefit to liver transplantation for HCC by an additional 5–20% to HCC patients who would have otherwise been excluded from liver transplantation under the Milan criteria [5]. In 2008, Toso et al., proposed the total tumor volume (TTV) concept. The TTV was calculated by adding the volume of each individual tumor, and a TTV cut-off of 115 cm^3^ was set for the risk of recurrence by using a receiver operating characteristic curve. While more patients qualify for a transplant with the TTV criteria (28–53% more than with the Milan criteria, and 16–26% more than with the UCSF criteria), no marked deterioration in the post-transplant outcome was demonstrated in patients with a TTV <115 cm^3^ compared with those meeting the Milan or UCSF criteria [6]. In addition, the radiological findings correlated closely with the post-transplant pathological diagnosis when using the TTV criteria than with the Milan or UCSF criteria (91% vs. 69% or 75% of patients, respectively). In 2009, Mazzaferro confirmed a socially acceptable prognosis (a 5-year survival rate of ≥70%) and analyzed the acceptable tumor burden of HCC lesions. That analysis showed that patients were able to achieve a 5-year overall survival rate of 71.2% as long as the HCCs were within “seven” as the sum of the size of the largest tumor (in cm), and the number of lesions (the “Up-to-7” criteria) [7]. The expanded morphological criteria for living donor liver transplantation (LDLT) were also reported as the Tokyo criteria in 2007 [8] and as the Asan criteria in 2008 [9] (representative criteria shown in Table 1).

## 3. Extended Criteria Incorporating the Biological Behaviors of HCC

Although attempts to expand the criteria have been made by increasing the diameter and number of HCC lesions, these two morphological parameters represent only a part of the biological behaviors of such tumors. In 2008, Zheng reported the Hangzhou criteria, which incorporated AFP as a surrogate marker for biological behaviors. They concluded that HCC patients could be considered eligible for liver transplantation when satisfying one of the following two points: (a) a total tumor diameter of ≤8 cm, or (b) a total tumor diameter of >8 cm but a histopathologic grade I or II and a preoperative AFP level of ≤400 ng/mL, since the survival curves of patients who met the Hangzhou criteria were statistically equal to those patients who met the Milan criteria [10]. This was the first report in which AFP was selected as a surrogate maker of HCC’s biological behavior. DuBay also reported that a protocol using a pathological diagnosis to exclude poorly differentiated tumors and an aggressive bridging therapy achieved excellent survival rates after liver transplantation, irrespective of the tumor size or the number of HCC lesions [11]. Since the publication of these reports, the biological behavior of HCC has been recognized as an indispensable factor that should be included in criteria that attempt to expand the list of HCC patients eligible for liver transplantation and to improve the post-transplant outcome.

Many previous reports have described the apparent relationship between microvascular invasion [12] and histological differentiation with the risk of post-transplant recurrence. Based on the fact that the AFP level correlates with the MVI and degree of differentiation [13], and the PIVKA-II (DCP) level correlates with the MVI and micro-intrahepatic metastasis [14], these two tumor markers have been frequently selected as surrogate markers of the biological behavior of HCC. In the last decade, their inclusion in the development of criteria has become mainstream.

In 2012, Duvoux reported an AFP model scored by the tumor diameter, number, and AFP level. In their report, patients with a score of ≤2 were indicated for transplantation (with a 5-year survival rate of 70%). Furthermore, they addressed the importance of AFP for predicting post-transplant recurrence, as the AFP level was able to predict patients with a poor prognosis despite meeting the Milan criteria (AFP > 1000 ng/mL) and a good prognosis despite not meeting the Milan criteria [15]. Hameed at UCSF also emphasized that patients with a high AFP level (>1000 ng/mL) could show a poor prognosis even when they met the Milan criteria and that the AFP level needed to be reduced to <500 ng/mL prior to transplant [16]. In 2015, Toso, who had reported the TTV criteria, updated their criteria by adding an AFP cut-off (<400 ng/mL) to improve the reliability (TTV + AFP criteria) [17]. Mazzaferro, who reported the Milan and Up-to-7 criteria, developed the Metroticket 2.0 model in 2018. According to this model, the overall 5-year survival rate reached 79.7%, and tumor recurrence could be more accurately estimated than with the Milan, UCSF, AFP model, or Up-to-7 criteria [18]. Costentin reported that the Harrell’s C-statistics for the Metroticket 2.0 model were significantly higher than those for the Milan criteria (*p* < 0.001), UCSF criteria (*p* < 0.001), Up-to-7 criteria (*p* < 0.001), and AFP model (*p* = 0.044) [19].

Several criteria in the field of LDLT have been reported, mainly from Japan and South Korea, where deceased donors are scarce. In 2007, Todo incorporated the PIVKA-II and AFP levels into established criteria and reported the AP criteria based on an analysis using a Japanese national database. In their report, using the AP criteria (AFP < 200 ng/mL and PIVKA-II < 100 mAU/mL), the 5-year survival rate reached 82.0% in total [20]. The University of Tokyo group who reported the 5-5 criteria also pointed out the importance of incorporating the AFP (<400 ng/mL) and PIVKA-II (<200 mAU/mL) levels into the criteria [21]. In line with these reports, a Kyoto University group reported the Kyoto criteria based on 136 patients’ data [22]. A Kyushu University group also reported the Kyushu criteria based on their analysis of 109 cases [23]. Although the Kyoto and Kyushu criteria were characterized by the sole use of PIVKA-II as a biological marker of HCC, the 5-year survival rates reached 87% and 80%, respectively. Likewise, the Asan Medical Center in South Korea also advocated the importance of two tumor markers (AFP < 150 ng/mL and PIVKA-II < 100 mAU/mL) [24]. In the same year, a model to predict tumor recurrence after living donor liver transplantation (MoRAL) score (11 × √PIVKA-II + 2 × √AFP), was reported from three major liver transplant institutions in South Korea. According to the report, patients with a score of <314.8 could be expected to have a good prognosis, even when their HCC exceeded the Milan criteria [25] (representative criteria shown in Table 2).

In almost all the newly developed criteria, surrogate markers for the biological behaviors of HCC have been integrated with the morphological limitations. AFP has mainly been selected in Western countries, while PIVKA-II is occasionally selected in Asia. Although the importance of including surrogate markers in criteria has been widely recognized, which surrogate marker should be selected and when and how the marker should be used remains debatable.

## 4. Prediction for HCC Recurrence and Survival after Transplantation

New attempts have also been made to predict the high-risk recurrence group, adapting the histopathological findings of the resected liver. In 2017, a four-year follow-up result of the application of a model of recurrence after the liver transplant (MORAL) score was reported [27]. With this score model, the tumor diameter and neutrophil-to-lymphocyte ratio [28], as well as the AFP level, were selected as preoperative determinants for the pre-MORAL score. Furthermore, the post-MORAL score was calculated based on histopathological tumor factors (tumor diameter and number, MVI, and tumor differentiation), and finally, the combo-MORAL score was estimated as the sum of the pre- and post-MORAL scores. According to the score, the patients were categorized into low, moderate, high, and very high-risk groups of post-transplant recurrence. The pre-, post-, and combo-MORAL scores better predicted HCC recurrence after liver transplantation than the Milan criteria, with C-statistics of 0.82, 0.87, and 0.91, respectively [27]. The RETREAT score, reported in 2018, also predicts the recurrence rate using the MVI from postoperative histopathological examinations in combination with the maximum tumor diameter, number of tumors, and AFP level [29]. In accordance with the result, the risk of recurrence was predicted to be <10% in those with a score of ≤3 but >20% in those with higher scores.

Other than the post-transplant recurrence, the prediction of survival should be considered when determining the indication of transplantation for HCC. The hazard associated with liver transplantation for HCC (HALT-HCC) score, reported in 2017 [30,31], was a predictive formula constructed based on a study of 420 cases from 2002 to 2014 and was validated with 13,717 cases in the United States during the same period. As per the result, the prognosis deteriorated in a stepwise manner as the score increased, and if the score exceeded 17, the prognosis was considered poor. In 2020, a validation study using a total of 4089 cases (in the US, Europe, and Japan) was reported, and the score was deemed able to be adopted regardless of the region after correcting the HALT-HCC score. Furthermore, it has been reported that the score was able to predict the poorly differentiated components and MVI of HCC more accurately than the Milan criteria, Metroticket 2.0 model, or MORAL score [32] (Table 3).

These can be helpful in clinical practice for making decisions regarding tailoring post-transplant immunosuppressive therapy: reducing immunosuppressants, switching to mTOR inhibitors from calcineurin inhibitors, and modifying the post-transplant follow-up protocol on a per-patient basis.

## 5. Candidates of Surrogate Markers for Biological Behavior of HCC

Morphologically small HCC may have aggressive biological features with an unfavorable post-transplant outcome, while patients with non-small tumors can have a low risk of HCC recurrence after liver transplantation. Therefore, the morphological criteria have evolved to incorporate the biological behavior of HCC.

In this context, both the MVI and tumor differentiation, especially a poor histologic grade, have been shown to be important pathological factors [33]. While a liver biopsy remains the gold standard for evaluating these tumor characteristics, a pre-transplant tumor biopsy often underestimates tumor differentiation due to the heterogeneity of HCC [34]. Microvascular invasion cannot be assessed in a biopsy specimen due to sampling bias. Even in a whole explanted or resected specimen, there are sometimes difficulties in evaluating this histological feature [35]. To predict these pathological components, many surrogate markers have been examined. In addition to the above-mentioned factors, the proliferation activity and metastatic potential of tumor cells, as well as the inflammatory or immunological status around the tumor influencing its progression, should also be considered. To refine the morphological selection criteria of liver transplantation for HCC, several surrogate markers that are related to the tumor biology have been integrated into the recent criteria described above. These markers can be categorized as serum tumor markers, systemic inflammatory biomarkers conditioning tumor progression, radiological characteristics related to the biological behavior of HCC, and the prediction of the micro-metastasis through liquid biopsy. Furthermore, evidence regarding the tumor responses to locoregional treatments as an imperative determinant has recently been accumulated.

### 5.1. AFP

AFP has been reported as a surrogate marker of tumor differentiation and vascular invasion [36]. It has also proven to be a useful biomarker for identifying patients at high-risk for HCC recurrence after liver transplantation, depending on the AFP cut-off [37,38]. Additionally, AFP has been selected as a surrogate marker of tumor biology and integrated into almost all criteria reported in the past decade, including the Hangzhou criteria, the French AFP model, the TTV + FP model, and the Metroticket 2.0 model. These facts underscore the value of AFP as a surrogate marker for HCC tumor biology and for predicting HCC recurrence after liver transplantation. Although consensus concerning the best cut-off value has yet to be achieved, the integration of tumor biology (including AFP) into the selection criteria was recommended with strong strength for both the deceased and LDLT in the working group report from the 2020 International Liver Transplantation Society transplant oncology consensus conference [39].

Regarding the mechanism underlying the relationship between AFP and tumor behaviors, several potential processes have been proposed. Among them, intracellular AFP has been reported to upregulate HCC cell proliferation directly by activating the proto-oncogene tyrosine-protein kinase Src and c-myc. Furthermore, this type of AFP is considered to accelerate the metastatic potential by enhancing adhesion molecules, such as cytokeratin-19 and the epithelial cell adhesion molecule (EpCAM), through phophoinositide-3 kinase (PI3K)/AKT activation [40,41]. Alternatively, extracellular AFP has been reported to not only induce tumor cell proliferation via the AFP receptor but to also provoke the apoptosis of dendritic cells, causing the inhibition of the anti-tumor immune system [40,41].

### 5.2. PIVKA-II (DCP)

The abnormal form of prothrombin induced by vitamin K absence (PIVKA-II, also known as DCP) is produced during the malignant transformation of hepatocytes. It upregulates the expression of angiogenic factors, such as an endothelial growth factor receptor and a vascular endothelial growth factor [42,43]. Up-regulation of PIVKA-II has been found to correlate with the degree of malignancy of HCC, as PIVKA-II positive tumors are characterized by an increased risk of intrahepatic metastasis, capsule infiltration, and MVI [12,44]. Furthermore, PIVKA-II-positive and AFP-negative tumors are aggressive, since they are usually large tumors with poor differentiation and positive vascular invasion [45,46]. Based on these findings, PIVKA-II is considered a useful biomarker for identifying patients at high-risk of HCC recurrence after liver transplantation.

In the working group report from the 2020 ILTS transplant oncology consensus conference, the integration of PIVKA-II (<7.5 ng/mL [<624 mAU/mL]) into the selection criteria was recommended with strong strength in LDLT [39]. Additionally, PIVKA-II has been incorporated into several criteria, such as the Kyoto criteria, Kyushu criteria, AP criteria, and South Korean MoRAL score, with or without the AFP value. However, despite the usefulness of PIVKA-II as a biomarker, the selection criteria of liver transplantation for HCC incorporating PIVKA-II have mainly been reported from Asian countries, possibly due to its limited clinical applicability. Although consensus regarding the best cut-off value has yet to be reached, the selection criteria might be made more reliable by adding PIVKA-II rather than including just AFP alone. In this context, the Mayo clinic reported that the hazard ratio of HCC recurrence after liver transplantation was 5.2 when both AFP ≥ 250 ng/mL and PIVKA-II ≥ 7.5 ng/mL were considered together, compared to 3.5 and 2.8 when applying PIVKA-II ≥ 7.5 ng/mL alone and AFP ≥ 250 ng/mL alone, respectively [47].

### 5.3. NLR

The NLR is an inflammatory marker that is determined by the ratio of peripheral blood neutrophils to lymphocytes. The relationship between the NLR and recurrence after liver transplantation among HCC patients was first demonstrated by Halazun in 2009, where patients who met the Milan criteria with an NLR of ≥5 had a poorer recurrence-free survival (RFS) than those with an NLR of <5 (25% vs. 75%) [48]. Since then, the NLR has been considered as a possible independent risk factor for post-transplant HCC recurrence. A meta-analysis conducted by Xu of 13 studies including 1936 transplanted HCC patients showed that an elevated NLR was associated with a decreased RFS after liver transplantation (hazard ratio (HR): 3.77). In addition, the study indicated that an elevated NLR was associated with the presence of vascular invasion (odds ratio (OR): 2.39) and was less frequent among patients who met the Milan criteria than in those who did not (OR: 0.26) [49].

The NLR is usually selected as a biological marker, not alone but rather in combination with other factors, such as the tumor size and number, and AFP or PIVKA-II levels. In this fashion, several patient criteria of liver transplantation for HCC have been proposed [50,51,52,53,54,55]. The NLR cut-off value varies among reports, ranging from 2.66 to 6.0. In this context, Xu recommended a cut-off of 4.0 based on the results of their meta-analysis in 2018 [49]. Shindoh assessed the ability of the NLR to predict post-transplant recurrence compared with the AFP and PIVKA-II values. After evaluating three different pre-transplant values (the value just before the transplant, and the maximum and mean values within 90 days before the operation) for the NLR, AFP, and PIVKA-II, the maximum AFP, and PIVKA-II values and the mean NLR were found to be independently associated with HCC recurrence. However, the NLR had a limited prognostic impact (AUC: 0.62), and the maximum AFP and PIVKA-II had sufficient predictive power (AUC: 0.88 and 0.76, respectively) [52]. In contrast, in the MORAL score proposed by Halazun in 2017, a preoperative NLR > 5 (HR: 6.2), AFP > 200 ng/mL (HR: 3.8), and tumor size > 3 cm (HR: 3.2) were found to be independently associated with a worse RFS, and it was concluded that the preoperative NLR was the most reliable predictive determinant of recurrence [27]. However, despite the NLR being potentially related to the post-transplant recurrence and outcome, conclusions regarding its consistency have not been reached by consensus. Variations might have been caused by differences in the profile of white blood cells between cirrhotic and non-cirrhotic patients.

The molecular mechanism underlying how the NLR is associated with HCC recurrence after liver transplantation has not been fully evaluated. However, it is hypothesized that neutrophils are involved in the vascular invasion and metastasis process by increasing the production of angiogenic factors, such as VEGF [56,57]. In contrast, it has been reported that IL-17 and tumor-associated macrophages (TAMs) rather than VEGF, contribute to tumor progression; an elevated NLR is correlated with the up-regulation of IL-17 and an increased TAM infiltration into peritumoral regions, by which HCC growth, tumor migration mediated by matrix metalloproteinase, and downregulation of the anti-tumor immune response are induced [28].

### 5.4. ^18^F-Fluorodeoxyglucose Positron Emission Tomography/Computed Tomography (^18^F-FDG PET/CT)

Regarding the radiological characteristics associated with the biological behavior of HCC, ^18^F-FDG-PET/CT has been attracting attention. In HCC, the growth rate, and activity of glycolytic enzymes are related [58]. In particular, poorly differentiated HCC cells have low glucose-6-phophatase activity and show high uptake of ^18^F-FDG [59]. Seo reported that the tumor/non-tumor ratio of the standardized uptake value [60] increased in a stepwise fashion according to tumor differentiation, with significant differences in the value noted between well- and moderately differentiated tumors and between moderately and poorly differentiated tumors [61]. Additionally, PET/CT indicated the biological status of the tumor aggressiveness and showed a good correlation with explant pathology in HCC. Furthermore, regarding the accuracy of PET/CT for detecting MVI and tumor differentiation, the range has been reported at 68.3–88.1% and 54.7–71.4%, respectively [62,63,64,65,66,67].

Seoul National University Hospital was the first to report the effectiveness of pre-transplant PET/CT for predicting post-transplant HCC recurrence [66]. Subsequently, Kornberg et al. reported that PET/CT-negative HCC patients might achieve an excellent post-transplant recurrence-free survival even when not meeting the Milan criteria [68]. Many studies have since reported a poor overall and recurrence-free survival in patients with PET/CT-positive HCC after liver transplantation. The recurrence-free survival rate at 3 years after liver transplantation was 35.0–57.1% in PET/CT-positive patients and 84.5% to 94.6% in PET/CT-negative patients [62,63,64,65,66,69]. When patients were divided according to meeting the Milan criteria, the gap in patients who did not meet the Milan criteria was increased compared to those who met the Milan criteria. Lee found the difference in patients who did and did not meet the Milan criteria to be 16.0% and 35.8%, respectively [62]. Based on these data, they concluded that PET/CT findings were more important in patients with advanced HCC than in others.

The usefulness of performing PET/CT in patients not meeting the morphological criteria was recently reported by a Taiwanese group who combined PET/CT results with the UCSF criteria (recurrence rate: 3.6% in those who met the UCSF criteria vs. 11.1% in those who did not meet the UCSF criteria with PET/CT negativity) [70], by a Korean group who incorporated PET/CT into the Milan and UCSF criteria (5-year recurrence-free survival rate: 73.3% in those who did not meet the Milan criteria with PET/CT negativity and 72.8% in those who did not meet the UCSF criteria with PET/CT negativity) [62], and by a German group [71] who applied the Up-to-7 criteria and PET/CT (5-year recurrence-free survival rate: 81.0% in those who met the Up-to-7 criteria vs. 87.1% in those who did not meet the Up-to-7 criteria with PET/CT negativity). In this context, a Japanese multi-center study showed that the subgroup of patients who did not meet the Milan criteria with negative PET/CT findings and a low AFP value achieved a 5-year recurrence rate of 19%, which was equivalent to that among patients who met the Milan criteria (7%, *p* = 0.1) [72]. Hong et al. further developed the concept, hypothesizing that the combination of the PET/CT and serum AFP level might improve the prediction of the post-transplant outcome for patients with HCC. Using a cut-off value of 200 ng/mL for AFP and 1.1 for the tumor-to-normal liver ratio maximum SUV value (SUV_max_ T/L ratio) to define “high-risk” HCC, they found that the rate of MVI and poor differentiation was 33% and 92%, respectively, in the high-risk group. Conclusively, the 5-year recurrence-free survival rate was only 8.4% in these high-risk patients [73].

The definition of PET/CT positivity has not reached a consensus due to the high uptake of ^18^F-FDG in the background liver. In this context, (a) the SUV_max_ of the tumor, (b) the SUV_max_ T/L, and (c) the ratio of the tumor SUV_max_ to the normal liver mean SUV (TSUV_max_/LSUV_mean_) have been recognized as objective indices for defining positivity. Among these, Lin found that the SUV_max_ T/L ratio was an independent predictor of vascular invasion and that the optimal cut-off values for the SUV_max_ of the tumor and SUV_max_ T/L ratio for predicting HCC vascular invasion were 3.80 and 1.49, respectively [74]. In contrast, Ahn reported the reliability of TSUV_max_/LSUV_mean_, where a value of >1.2 had a statistically significant association with MVI, with an odds ratio of 14.2 [75].

Although the definition of its positivity and the proper scope of patients to whom PET/CT should be applied remain matters of debate, PET/CT is still a potential candidate surrogate marker for incorporation into the selection criteria in a cost-benefit balance. In the working group report from the ILTS transplant oncology consensus conference, the integration of PET/CT into the selection criteria was recommended with strong strength in LDLT [39].

### 5.5. Liquid Biopsy

Liquid biopsy has attracted much attention as a possible surrogate marker for the biological behavior of HCC. Circulating tumor cells (CTCs) or tumor cell-free nucleic acids in peripheral blood have mainly been utilized as an index of micro-metastasis. Li et al., reported their utility in diagnosing and prognosis prediction of HCC [76]. Since the liver is thoroughly removed in LDLT or deceased donor liver transplantation, post-transplant HCC recurrence is not theoretically encountered in the absence of extrahepatic micro-metastasis. Meanwhile, circulating HCC cells have not always been associated with post-transplant HCC recurrence, even when they are detected preoperatively [77]. Xue et al., has reported its usefulness as a good prognostic indicator for post-transplant HCC recurrence in which an immunostaining-fluorescence in situ hybridization (iFISH) platform was used to detect CTCs. They concluded that preoperative iFISH-CTCs ≥ 5/7.5 mL was the most valuable determinant for recurrence-free survival after liver transplantation [78]. Wang et al., also reported that a postoperative CTC measurement was a potential biomarker for predicting tumor recurrence after transplantation and emphasized the importance of postoperative serial CTC detection in surveillance to detect the recurrence of HCC [79]. However, very little data are available to support the potential role of CTCs as a preoperative predictor of the recurrence of HCC after liver transplantation.

In terms of circulating cell-free nucleic acids, the AFP mRNA expression in peripheral blood has been suggested as a surrogate of CTCs. Marubashi et al. reported its usefulness in predicting the recurrence of HCC after LDLT [80]. However, the utility is controversial, and some authors even consider AFP mRNA to be non-specific for micro-metastasis of HCC.

Although the complex methods used to detect these tumoral components have improved dramatically, a consensus has yet to be obtained with regard to the methodology, and the improvement of sensitivity remains an issue to be resolved. Furthermore, evidence of their association with HCC recurrence after liver transplantation should be proven in a large multi-center cohort. Thus, at the present time, no firm conclusion has been drawn regarding the value of a preoperative liquid biopsy for predicting post-transplant recurrence of HCC.

### 5.6. Response to Locoregional Therapy

To understand the actual biological tumor behavior, the tumor dynamics should be evaluated, rather than consulting a snapshot tumor marker or inflammatory biomarker value or the radiological characteristics. Assessing the response to locoregional therapies, such as transarterial chemoembolization (TACE), selective yttrium-90 radioembolization (Y-90) and/or radiofrequency ablation (RFA), can help clarify the individual tumor behavior based on dynamic changes. A single-center experience in Germany demonstrated that HCC patients who were free from progression after TACE achieved an excellent 5-year RFS rate post-transplantation compared to patients with progressive disease (PD) after initial TACE [81]. Furthermore, the complete response (CR) rate promoted by TACE led to a superior 5-year RFS rate (94.4%), whereas the partial response (PR) and stable disease (SD) were associated with lower RFS rates (46.7% and 50.0%, respectively) [82]. In line with this, the rate of post-transplant HCC-related death (5-year) was significantly lower in those with CR to pre-transplant locoregional therapy (n = 355, 3.1%) than in those with PR/SD (n = 214, 9.6%) or PD (n = 290, 13.4%) [83]. Similarly, when locoregional treatment resulted in CR or PR, the incidence of waitlist dropout and recurrence rate was dramatically reduced [84,85,86]. Recent data from the US Multicenter HCC Transplant Consortium (UMHTC) that included 3439 adult patients who received pre-transplant locoregional therapies revealed that 802 (23%) patients with HCC achieving pathological CR (pCR) on explant pathology had a superior 5-year RFS rate (73%) compared to those without pCR (n = 2637, 64%). The study identified predictive factors associated with pCR as being a low AFP value, low NLR, female gender, low MELD, and young age. Meanwhile, the factors being less likely to achieve pCR were HCV and cryptogenic cirrhosis, a short wait-time region, and multiple locoregional therapies (≥3) [87]. Additionally, despite locoregional therapy, an AFP slope >7.5 ng/mL per month was associated with HCC recurrence post-transplantation (HR, 3.0, *p* = 0.03) [88]. A study of LDLT from a Kyoto group showed that the number of pre-transplant treatments, including resection and locoregional therapies, could predict the risk of HCC recurrence post-transplantation, with patients who had undergone ≥5 pre-transplant treatments showing a higher 5-year recurrence rate than others (30% vs. 7% no pre-transplant treatment, *p* = 0.09) [89]. Thus, the response to locoregional therapy is closely correlated with post-transplant HCC recurrence.

The response to locoregional therapies likely serves as a useful surrogate marker for predicting HCC recurrence after liver transplantation [90]. Indeed, accumulated evidence shows that the radiographic response to locoregional therapy should be included in the selection criteria for optimizing survival post-transplantation. In this context, it has been reported that the Metroticket 2.0 framework should be adjusted by modified Response Evaluation Criteria in Solid Tumors (mRECIST) [83]. In brief, some cases of PR/SD and all cases of PD should be applied with the modified Metroticket 2.0 framework corresponding to the mRECIST grade to accomplish a cumulative incidence of HCC-related death of <30% [83]. Furthermore, the time-radiological-response-alpha-fetoprotein-inflammation [TRAIN] score which is determined by radiological response to locoregional therapy evaluated by mRECIST, AFP slope, the NLR, and waiting time has emerged to guide transplantable patients [91]. A retrospective multi-center study revealed that changes in the HALT-HCC score during locoregional therapies could stratify the risk of waitlist dropout and recurrence post-transplantation [92]. The HALT-HCC score as well as the TRAIN score, which is a longitudinal evaluation of the tumor dynamics, may quantify the individual risk more effectively than a category-based evaluation, such as mRECIST. The working group of the ILTS noted that composite criteria, including the response to locoregional treatments, are likely to replace conventional morphological criteria for defining transplant feasibility [39]. It is conceivable that observing the tumor behavior after locoregional therapies would provide valuable insight into selection criteria for precisely predicting the risk of post-transplant recurrence of HCC, although there is a limitation in that locoregional therapy is only applied to patients with a well-preserved liver function. The most accurately predictive criteria for post-transplant HCC recurrence may thus depend on the patient’s oriented algorithm.

## 6. Selection Criteria and Allocation Rule Worldwide

The significance of extended criteria concerns maximizing each patient’s benefit, and the argument concerning selection criteria is based on scientific fact. However, an allocation system cannot be established by scientific fact alone, as social situations should be taken into consideration. This is because the number of available donations and the distribution of liver disease etiology vary markedly among countries [93]. Therefore, an allocation system should be implemented based on both scientific facts and a given country’s situation.

There are three major principles concerning allocation rules: urgency, utility, and transplant benefit [94]. Medical urgency is the most widely accepted principle and has been widely used. The “sickest first” policy approach is useful for saving patients who would otherwise die without liver transplantation. To reduce the waiting list mortality, an approach based on medical urgency is considered the best allocation policy. As the medical urgency is estimated by the MELD score, it is difficult to incorporate HCC factors into an urgency-based system, as the disease condition varies widely, represented by biological markers such as AFP, PIVKA-II, and the NLR, even in T2 patients. In this context, individual prediction models, such as the HALT-HCC [30], can precisely reflect HCC patients’ medical urgency. However, no definite solution concerning how HCC factors should be integrated into the MELD score has yet been proposed. Therefore, many countries treat HCC patients as a homogenous group and award extra points depending on their time on the wait list, which reflects the tumor progression.

The concept of utility involves maximizing limited donations and avoiding wasting available grafts [95]. This system allocates livers to recipients with geographic advantages (i.e., based on proximity) or assigns a priority of patient selection to transplant centers (center-oriented allocation). While wasting of limited donor organs may be reduced with this approach, waiting list mortality may increase due to the avoidance of futile transplantation, as “the sickest patient” is not an ideal candidate in this system. To address this imbalance, several models have been developed to adjust donor-recipient matching, such as the D-MELD [96], SOFT [97], and BAR scores [98]. These models help to ensure equity by improving donor-recipient matching—although HCC factors are difficult to integrate into this system. Therefore, a medical-urgency system involving special HCC scores has been the mainstream national allocation system for HCC patients.

The new concept of “transplant benefit” [99] was developed to ensure equity among heterogeneous patients on the waiting list [100]. In brief, transplant benefit refers to how much lifetime is gained by liver transplantation (“gain in life expectancy”), expressed as the difference between the estimated lifetime without liver transplantation and the survival after liver transplantation. This new concept enables both HCC and non-HCC patients to be evaluated equally, and a transplant-benefit based allocation system is now implemented as the national allocation policy in several countries.

Table 4 shows the patient selection criteria and allocation system used to prioritize HCC patients in each country. With the accumulation of cases, several extended criteria have become used as national selection criteria, and each country has developed its own allocation system.

In the United States, for example, the chance to undergo liver transplantation is offered to all patients with HCC. However, extra MELD points are given to T2 patients, as long as the AFP is ≤1000 ng/mL [101]. Based on national data reporting that the prognosis of T2 HCC patients at the registry is similar to that of patients with a MELD score of 22, the MELD score starts at 22 in T2 HCC patients, and extra points are assigned in a time-dependent manner, reflecting disease progression (2 points every 3 months).

In Canada, the selection criteria and allocation rules differ among provinces. As the selection criteria, the Milan criteria are used in British Columbia; the TTV + AFP model in Alberta, Ontario, and Nova Scotia; and the Milan criteria or TTV + AFP model in Quebec. The conditions for assigning extra points also vary among provinces, as shown in Table 4. Efforts are underway to establish a national consensus for allocation criteria [102].

In the United Kingdom, as long as the AFP value is ≤1000 ng/mL, HCC patients with ≤5 nodules that are ≤3 cm in diameter or a single tumor of >5 cm but ≤7 cm in diameter with no evidence of tumor progression over a 6-month period are allowed to register with the waiting list [103]. Bridging locoregional therapy is permitted for all patients while awaiting transplantation, but a down-staging policy has yet to be established. Regardless of the tumor burden, no extra points are assigned.

Euro-transplant, excluding Germany, has adopted the Milan criteria as the selection criteria. For HCC patients to be added to the wait list, one of the following is required: (a) biopsy-proven HCC, (b) an AFP level of ≥400 ng/mL and one positive result with an imaging technique, or (c) two positive results with imaging techniques. Extra points are given to HCC patients both at listing and every 90 days thereafter [104].

In Spain, the Milan criteria have been considered the standard selection criteria. Patients with an AFP level of >1000 ng/mL should undergo locoregional therapy, and those with an AFP level of <500 ng/mL are required to be included in the waiting list. Recently, a moderate expansion was carried out and the Up-to-7 criteria were added to the selection criteria. Patients beyond the Milan criteria but within the Up-to-7 criteria are required to have an AFP level of <400 ng/mL. If their AFP level is ≥400 ng/mL, these patients should undergo locoregional therapy, and be re-evaluated after 1 month [105].

In France, their original prediction model—the AFP model [13]—has been implemented as the national selection criteria since 2013. HCC patients with an AFP model score of ≤2 are eligible for liver transplantation. France’s allocation rule consists of an original point system in which the points at registration for HCC patients are determined according to the MELD score, and extra points are added in a time-dependent fashion. To ensure utility, the gravity model, which incorporates the distance between the donor and recipient locations into the HCC point total, is adopted in the allocation system [106].

In Italy, HCC patients can be included on the wait list so long as a 5-year survival rate of 50% is expected, and multiple criteria (e.g., Milan criteria, UCSF criteria, TTV criteria, Up-to-7 criteria, French AFP model, etc.) are allowed to be used [107]. The Italian allocation policy is unique and characterized by a transplant-benefit based system. Their system evaluates HCC and non-HCC patients according to the 5-year transplant benefit using the HCC-MELD score [100]. HCC-MELD creates a “MELD equivalent”, which matches HCC patients with non-HCC patients who have the same MELD score so that equity is ensured.

In South Korea, the Milan criteria are used as selection criteria. Based on their nationwide data [108], the 90-day outcomes were similar between HCC patients with a MELD score of <14 and non-HCC patients with a MELD score of 14–17; therefore, an additional 4 points are given to HCC patients with a MELD score of <14. In addition, the waiting list survival rates were similar between HCC patients with a MELD score of 14–20 and non-HCC patients with a MELD score of 21–25, so an additional 5 points are given to HCC patients with a MELD score of 14–20.

In Japan, where LDLT is the mainstay for those requiring liver transplantation, the Milan criteria are the government-approved criteria for national insurance coverage for patients with HCC, aiming at restricting the undue expansion of indications for advanced HCC. However, some centers have proposed and utilized center-oriented extended indications, such as the Kyoto criteria, for those wishing to undergo LDLT in a private practice setting. Therefore, there has been a strong demand and movement toward expanding the nationwide-approved criteria, as in other countries. The goal of the new extended criteria for HCC patients would be to enable the maximal enrollment of candidates while securing a 5-year recurrence rate below 10% (with a 95% upper confidence limit) by examining various combinations of tumor numbers and AFP and/or PIVKA-II values, maintaining the maximal tumor diameter at 5 cm. An analysis was conducted based on retrospective data from a Japanese nationwide survey of LDLT, including 965 patients undergoing LDLT, 301 of whom did not meet the Milan criteria. Ultimately, the 5-5-500 criteria (tumor ≤ 5 cm in diameter, tumor number ≤ 5, and AFP ≤ 500 ng/mL) were established, with a 5-year recurrence rate of 7.3% (with a 95% confidence interval at 5.2–9.3) and a 5-year survival rate over 70% [26]. Since the exclusion of patients from liver transplantation with these new criteria who otherwise met the Milan criteria did not seem socially acceptable or rational, patients with HCC who met the 5-5-500 or Milan criteria are considered candidates for liver transplantation. By adopting these double eligible criteria, a 19% increase in the number of eligible patients over the number deemed eligible with the Milan criteria alone was achieved. Although the 5-5-500 criteria were established in a living donor setting, the new double eligible criteria have become nationally applied in Japan for deceased donor liver transplantation in August 2019, and for the health insurance-covered LDLT in April 2020. The allocation system involves the MELD score in Japan, and extra points are awarded to HCC patients in a constant manner (2 points for every 3 months on the waitlist).

As described in this section, the discrepancy between the number of liver donations and that of transplant candidates markedly influences the differences in allocation rules among countries. However, these rules should aim at maximizing the transplant benefit of all patients who need liver transplantation. To this end, the selection criteria and allocation rules of liver transplantation for HCC patients should be meticulously revised based on the social situation and clinical status.

## 7. Down-Staging or Bridging to Liver Transplantation

In addition to the extension of criteria, the concept of down-staging can confer an additional opportunity for HCC patients to receive liver transplantation. Indeed, both single- [109,110,111] and multi-center [112] studies have reported a favorable RFS rate post-transplantation in patients who successfully achieved down-staging with locoregional therapies to meet the eligibility criteria. A study from UCSF showed that both the 5-year post-transplant survival and RFS rate were comparable between T2 patients and those in the down-staging group [109]. A group from the University of Bologna showed that patients who achieved down-staging to meet the Milan criteria had a comparable RFS rate (1 year: 78%, 3 years: 71%) to those who initially met the Milan criteria (1 year: 80%, 3 years: 71%) [110]. However, recent data from the US Multicenter HCC Transplant Consortium (20 centers) revealed a significantly higher HCC recurrence rate post-transplantation (5 years: 18.7%) in the patients who achieved down-staging to meet the Milan criteria (n = 465) than in those who initially met the Milan criteria (11.1%, n = 3570) [113]. This suggests that the previous study from Region 5 in the US, with a longer wait-time until liver transplantation, may lead to the selection of patients with more favorable tumor biology than others. In turn, these studies support the notion that biological tumor behavior in response to down-staging treatment may be a surrogate marker for predicting post-transplant HCC recurrence, as described in the Section 5.6. Thus, down-staging treatment has been recognized as an acceptable tool for certain patients with HCC who do not meet the conventional criteria.

The keys to down-staging treatment are as follows: (1) eligibility criteria; (2) type of locoregional therapy; (3) endpoint; (4) minimum observation period; and (5) exclusion criteria [114]. 1. Eligibility criteria. The eligibility criteria for down-staging were defined in the UCSF down-staging protocol as a national policy in the US. The UCSF down-staging protocol was classified as meeting one of the following criteria: a single lesion of ≤8 cm, 2 or 3 lesions each at ≤5 cm with the sum of the maximal tumor diameter being ≤8 cm, and 4 or 5 lesions each at ≤3 cm with the sum of the maximal tumor diameter being ≤8 cm [109], in which 65% of patients who met the criteria successfully converted to the Milan criteria, with only 7.5% showing post-transplant HCC recurrence [109]. A multi-center (3 centers in California) cohort study, according to the UCSF down-staging protocol, showed an excellent RFS rate post-transplantation (1 year: 95.4%, 3 years: 87.3%) [112]. Furthermore, the UNOS database, as a national experience of the US, showed that the 3-year survival rate post-transplantation was 83.2% in the group that always met the Milan criteria (n = 3276), while 79.1% were in the successfully down-staged group whose HCC initially met the UCSF inclusion criteria (n = 422, *p* = 0.17 vs. who always met the Milan criteria), and 71.4% were in the successfully down-staged patients who initially had not met the UCSF inclusion criteria (n = 121, *p* = 0.04 vs. who always met the Milan criteria) [15]. More recently, a prospective multiregional study (7 centers, 4 UNOS regions) also showed that over 80% of patients successfully underwent down-staging to meet the Milan criteria, with a low HCC recurrence rate (7.9%) following the UCSF down-staging protocol [115]. The findings described above support the value of the UCSF down-staging protocol as a national policy in the US. However, when down-staging without an up-front stage restriction (i.e., down-staging for “all-comers”), the successful down-staging rate decreased to 42.4%, although the post-transplant RFS rates of patients with any stage (except for stage IVB) who successfully down-staged to meet the Milan criteria (n = 63) were comparable to those in the patients who initially met the Milan criteria [116]. Although restricting the down-staging protocol was recommended in a consensus meeting [39], a national policy for a down-staging protocol has only been precisely defined in the US. 2. Type of locoregional therapy. Locoregional therapies for down-staging usually include alcohol injection, RFA, TACE, TARE, or liver resection [117]. The indication for locoregional therapy obviously carries safety concerns. The guidelines for TACE propose it be applied to cases of Child A/B and T-bil ≤ 3 mg/dL [118]; otherwise, there is a risk for acute hepatic decompensation, which renders a patient ineligible for liver transplantation [109]. 3. Endpoint. The endpoint of down-staging is defined as a reduction in the viable tumor burden to meet the acceptable criteria for LT, usually the Milan criteria. Other studies define a response to locoregional therapy based on the mRECIST criteria. In addition, patients with an AFP ≥ 400 ng/mL in the Bologna group and an AFP >1000 ng/mL in the US are considered to be excluded from LT [114]. 4. Minimum observation period. A minimum observation period of 3 months after successful down-staging is widely recommended [117]. 5. Exclusion criteria. Tumor progression with the development of macro-vascular invasion or extrahepatic spread is obvious exclusion criteria for down-staging and LT [114].

Locoregional therapy is also performed as a bridging therapy to reduce tumor burden while awaiting transplantation. Additionally, locoregional therapy as a bridging therapy has been recognized as being important given the long waiting time until a transplant, and it can endow a favorable outcome post-transplantation. The ILTS working group and AASLD guidelines noted that bridging therapy to prevent waitlist dropout seemed justified when the waiting time was expected to exceed six months [39,119]. However, in cases with small tumors (i.e., T1), bridging therapy does not affect the recurrence rate or overall survival [111]. Either way, bridging therapy with an objective response may indicate a patient is likely to have a favorable outcome post-transplantation.

Furthermore, recent developments in systemic therapy, including molecular-targeted treatment or immunotherapy, are being tested as bridging therapy [120]. Despite findings only being reported for a small number of cases (n = 9), the successful effect of nivolumab in one-third of patients with pCR and explant pathology was recently demonstrated [121]. Furthermore, promising evidence concerning immune checkpoint inhibitors in advanced HCC cases is expected to lead to the development of new treatment protocols as a bridging therapy in the future.

## 8. Discussion

The Milan criteria have heralded in a new era of liver transplantation as being recognized as a reliable treatment of choice for HCC. Since the establishment of the Milan criteria, the liver transplant community has been committing itself to expand indications for patients who would benefit from this procedure. The expansion of eligibility was initially attempted by means of raising the threshold of morphological tumor burden from that described in the Milan criteria. As data accumulated, the morphological criteria shifted to include the biological behaviors of tumors, aiming at a more reliable prediction of post-transplant recurrence. As surrogate markers for biological behaviors, serum tumor markers, systemic inflammatory biomarkers, and radiological characteristics have frequently been proposed. However, which of these is the best choice has not yet been conclusively determined. Furthermore, how each determinant should be weighed, how they should be combined, and to whom they should be applied also remains the subject of debate. Recently, the tumor dynamics, including the response to locoregional therapy, have garnered greater attention than the situational snapshot obtained by simply evaluating tumor characteristics. Although the criteria have evolved from a dichotomous nature to categorization (stepwise manner) and further, to prediction models scoring the risk of recurrence, composite criteria that combine surrogates for tumor biology and the tumor dynamics might replace conventional morphology-based criteria for selecting patients with HCC for liver transplantation.

The patient selection criteria must be based on evidence from the post-transplant recurrence and overall outcome. In LDLT, the selection criteria directly regulates the eligibility by balancing the recipient benefit against the donor risk. In consideration of the selection criteria for HCC in LDLT, the ethical consideration is to justify the risk of living donation if the probability of 5-year survival is expected to be ≥60%, as described in a recent ILTS consensus statement [39]. Allocation rules in deceased donor liver transplantation should be employed while considering the social situation as well, not least of all because of the balance between the number of organs donated and candidates and the distribution profile of HCC and non-HCC patients on the waiting list differ markedly among countries. However, no matter the situation, the concept of transplant-benefit must be borne in mind, and further refinement with the accumulation of data is imperative.

The down-staging concept is another promising way to expand the pool of HCC patients who would benefit from liver transplantation. However, whether the concept can be applied to all-comers with HCC or if it should be restricted to those with a certain tumor burden remains controversial. In this context, the recent development of systemic treatments for HCC, such as tyrosine kinase inhibitors—including lenvatinib—and immune checkpoint inhibitors, may provide evidence regarding the limitations of this approach.

## 9. Conclusions

Through the paradigm change in the selection criteria for liver transplantation for HCC, from solely morphological to the incorporation of surrogate markers for the biological behaviors, the acceptable tumor burden has gradually been clarified and the patients who can be expected to benefit from this procedure can be appropriately selected. To improve the thresholds of liver transplantation for HCC, one goal is to increase the number of eligible patients. The number of deceased donors, as well as the allocation rule, would directly influence the expansion of HCC patients who are eligible for liver transplantation. In terms of the tumor burden, early tumor detection is indispensable. Further, pre-transplant bridging therapy to prevent dropout due to tumor progression beyond selection criteria, and down-staging to an acceptable tumor burden in those who would otherwise be excluded from candidacy would contribute to the evolution of the thresholds. Another strategy for improvement is the further expansion of the selection criteria. In this regard, effective perioperative adjuvant therapy based on recent developments in anti-cancer drugs and the refinement of post-transplant immunosuppression regimens would play a key role.

## Figures and Tables

**Table 1:** Extended criteria by tumor morphology (diameter and number of HCC).

Criteria	Author	Year	Donor Setting	Institution	Criteria	Cases	Outcome	External Validation
Milan	Mazzaferro [3]	1996	DD	Univ. of Milan, Italy	Single tumor < 5 cm	48	4-year survival rate: 75%	◯
					Up to 3 tumors with diameter < 3 cm			
UCSF	Yao [4,5]	2001, 2007	DD	Univ. of California, USA	Solitary tumor < 6.5 cm	168	5-year survival rate: 75.2%	◯
					< 3 nodules with the largest lesion < 4.5 cm and total tumor diameter < 8 cm			
Total Tumor Volume (TTV)	Toso [6]	2008	DD	Univ. of Alberta, Canada	TTV less than 115 cm^3^	228	Within Milan: 5-year survival rate: 82%	◯
							Within TTV: 5-year survival rate: 80%	
Up-to-7	Mazzaferro [7]	2009	DD	International multicenter	HCCs with seven as the sum of the size of the largest tumor [in cm] and the number of tumors	1556	Within Milan: 5-year survival rate: 73.3 %	◯
							Within Up-to-7: 5-year survival rate: 71.2%	
Tokyo	Sugawara [8]	2007	LD	Univ. of Tokyo, Japan	HCC diameter: 5 cm or less, HCC number: 5 or less	78	5-year survival rate: 75%	◯
Asan	Lee [9]	2008	LD	Asan Medical Center, Korea	HCC diameter 5 cm or less, HCC number 6 or less	229	5-year survival rate: 76%	◯

DD: Deceased donor, LD; Living donor, ◯: Externally validated.

**Table 2:** Extended criteria by adding the surrogate markers for biological behavior of HCC.

Criteria	Author	Year	Institution	Criteria	Cases	Outcome	External Validation
Hangzhou	Zheng [10]	2008	Zhejiang University, China	Total tumor diameter less than or equal to 8 cm	195	Within Milan: 5-year survival rate: 78.3%	◯
				Total tumor diameter more than 8 cm, with histopathologic grade I or II and preoperative AFP level less than or equal to 400 ng/mL		Within Hangzohu: 5-year survival rate: 72.3%	
Toronto	Dubay [11]	2011	Univ. of Toronto, Canada	No vascular invasion on imaging studies	294	Within Milan: 5-year survival rate: 72%	
				HCC is confined to the liver, and not poorly diffentiated on biopsy.		Within Tronto: 5-year survival rate: 70%	
AFP model	Duvoux [13]	2012	French Study group, France	HCC size (cm): ~3 (0)/3.1~6 (1)//6.1~ (4)	537	Less than score 2: 5-year survival rate: 70%	◯
				Number of HCC: ~3 (0)/4~ (2)	435 (validation)		
				AFP (ng/mL): ~100 (0)/101~1000 (2)/1001~ (3)			
TTV+AFP	Toso [17]	2015	Univ. of Alberta, Canada	TTV less than 115 cm^3^	233	Within TTV/AFP but beyond Milan: 4-year survival rate: 74.6%	
				AFP less than 400 ng/mL			
Metroticket 2.0 model	Mazzaferro [18]	2018	Multicenter, Italy	Up-to-7 & AFP < 200 ng/mL	1018	5-year survival rate: 79.7%	◯
			Fudan Univ., Chila	Up-to-5 & AFP 200–400 ng/mL	341 (validation)		
				Up-to 4 & APP 400–1000 ng/mL			
AP criteria	Todo [20]	2007	Multiceter, Japan	AFP (<200 ng/mL) and PIVKA-II (<100 mAU/mL) to the Milan criteria	653	5-year survival rate: 82.0%	
Kyoto	Takada [22]	2007	Univ. of Kyoto, Japan	Maximum diameter of < 5 cm, <10 tumors, and PIVKA-II < 400 mAU/mL	136	5-year survival rate: 87%	◯
Kyushu	Shirabe [23]	2011	Univ. of Kyushu, Japan	PIVKA-II < 300 mAU/mL, regardless of the number of tumors, as long as it is less than 5 cm in diameter	109	5-year disease free survival rate: 80%	
MoRAL score	Lee [25]	2016	Multicenter, Korea	MoRAL Score (11 × √PIVKA-II + 2 × √AFP) < 314.8	566	Low Moral but beyond Milan: 5-year survival rate: 82.6%	◯
Japan	Shimamura [26]	2019	Multicenter, Japan	Nodule size < 5 cm in diameter, nodule number < 5, and AFP < 500 ng/mL	965	Within 5-5-500: 5-year overall survival rate: 75.8%	
						Within Milan or 5-5-500: 5-year survival rate: 74.8%	

◯: Externally validated.

**Table 3:** Prediction for HCC recurrence and survival after transplantation.

Criteria	Author	Year	Institution	Risk Factors	Cases	Cut-Off	External Validation
MORAL score	Halazun [27]	2017	Weill Cornell Medical college, USA	Pre-MORAL: Max size > 3 cm (3), AFP ≥ 200 ng/mL (4), NLR ≥ 5 (6)	339	Low risk ≤ 2	
				Post-MORAL: Grade 4 tumor (6), Vascular invasion (2), Max size > 3 cm (3), Number > 3 (2)		Mod. risk 3–6	
				Combo-MORAL: Pre-MORAL+Post-MORAL		High risk 7–10	
						very High risk >10	
RETREAT score	Mehta [29]	2018	Univ. of California, USA	Max size + Number: 0 (0)/1~4.9 (1)/5~9.9 (2)/≥ 10 (3)	3276	3-year recurrence rate	◯
				AFP (ng/mL): 0~20 (0)/21~99 (1)/100~999 (2)/≥ 1000 (3)		Score 0 = 1.6%	
				Presence of microvascular invasion: − (0)/+ (2)		Score 1 = 5.0%	
						Score 2 = 5.6%	
						Score 3 = 8.4%	
						Score 4 = 20.3%	
						Score 5 ≤ 29.0%	
HALT-HCC	Sasaki K [30]	2017	Cleveland Clinic	HALT-HCC score = 1.27 × (TBS (tumor burden score)) + 1.85 × 1n (AFP) + 0.26 × (MELD-Na)	420	5-year overall survival	◯
			SRTR		13,717 (validation)	Q1: 78.7%	
						Q2: 74.5%	
						Q3: 71.8%	
						Q4: 61.5%	
Recalibrated HALT-HCC	Firl DJ [32]	2020	4 centers in North America	Recalibrated HALT-HCC score = 1.33 × TBS + 2.31 × 1n (AFP) + 0.25 × (MELD-Na) − (5.57 in Asia)	4089	lowest-risk patients (HALTHCC 0–5)	
			10 centers in Europe			highest-risk patients (HALTHCC > 35)	
			2 centers in Asia				

◯: Externally validated.

**Table 4:** Allocation and Prioritization rules.

Country	Allocation Rule	Selection Criteria	Exception Point	Down Staging
		Criteria for Exception Points		
United States [101]	Patient-oriented	All comers	Start at 22, increase by 2 points every 3 months	Yes
		One lesion ≥ 2 cm and ≤ 5 cm, 2 or 3 lesions 1 cm and ≤ 3 cm		
		AFP level ≤ 1000 ng/mL		
Canada [102]	Provincial	British Columbia uses the Milan criteria. In Alberta, Nova Scotia and Ontario, total tumor volume (TTV) and AFP are used as selection criteria. In Quebec, Milan or TTV and AFP are used.	British Columbia: start at 15, increase by 3 points every 3 months	Yes
			Alberta: MELD-Na for 6 months, then 26 points: increase by 2 points every 3 months	
			Ontario: start at 22, increase 3 points every 3 months	
		British Columbia: lesion > 2 cm	Nova Scotia: MELD or assign 22 points	
		Alberta: lesion > 2 cm or Multiple lesion or recurrence after ablation		
		Ontario: same as Alberta		
		Quebec: If 1 tumor > 2 cm 16–25 points depending HCC characterics, or 25 points if TTV ≤ 115 cm^3^ and AFP ≤ 400 ng/mL		
		Nova Scotia: Lesion > 2 cm or multiple lesion		
United Kingdom [103]	DBD: patient-oriented	Single tumor ≤ 5 cm, up to 5 tumors all ≤ 3 cm, single tumor > 5 cm and ≤ 7 cm and no tumor progression over 6 months, and AFP ≤ 1000 ng/mL	No	No
	DCD center-oriented	No		
Eurotransplant [104]	Patient-oriented: G/B/N/L	Milan criteria, AFP level ≥ 400 ng/mL (except for Germany)	Initial exceptional MELD 15% of the 90-day predicted mortality, upgrade in 90-day steps +10%	
	Center-Oriented: A/H/S/C	Milan criteria, AFP level ≥ 400 ng/mL (except for Germany)		
Spain [105]	Center-Oriented	Milan criteria and AFP level ≤ 500 ng/mL, or Up-to-7 criteria and AFP level ≤ 400 ng/mL	No	No
France [106]	Patient-oriented	AFP score ≤ 2	MELD score at registration between 6 and 32, the number of points progressively increases up to a maximum ranging from 650 to 800 points	Yes
		HCC TNM ≥ 2 and AFP score ≤ 2	MELD score between 33 and 40, allocation only depends on MELD	
Italy [107]	Center-oriented	Either conventional (Milan criteria) or extended criteria (e.g., up to 7, total tumor volume, UCSF, a fetoprotein model) may be used to characterize a tumor as Transplantable, if they satisfy a minimal posttransplant utility requirement (50% 5-year patient survival)	TT_DR_-TT_PR_: HCC-MELD + extra points for time or MELD 22 at entry + extra points for time	Yes
		TT-HCC: any HCC meeting transplantability criteria (either conventional or expanded criteria)	TT_FR_: HCC-MELD Criteria for awarding extra points for longer waits and priority class migration on disease progression will be set regionally	
South Korea [108]	Patient-oriented	T2 tumor (Milan criteria)	MELD < 14 receive additional 4 points	
		T2 tumor (Milan criteria)	MELD between 14 and 20 receive additional 5 points	
Japan [26]	Patient-oriented	Milan criteria or 5-5-500	Exceptional 2 points is added to MELD score at registry every 3 months	Yes
		Milan criteria or 5-5-500		

G: Germany, B: Belgium, N: the Netherlands, L: Luxembourg, A: Austria, H: Hungary, S: Slovenia, C: Croatia, TT: Transplantable, TT_DR_-TT_PR_: downstaged patients or partial response to bridge, TTFR: first presentation or late recurrence.

## References

[1] Iwatsuki S., Starzl T.E., Sheahan D.G., Yokoyama I., Demetris A.J., Todo S., Tzakis A.G., Van Thiel D.H., Carr B., Selby R. (1991). Hepatic resection versus transplantation for hepatocellular carcinoma. Ann. Surg..

[2] Bismuth H., Chiche L., Adam R., Castaing D., Diamond T., Dennison A. (1993). Liver resection versus transplantation for hepatocellular carcinoma in cirrhotic patients. Ann. Surg..

[3] Mazzaferro V., Regalia E., Doci R., Andreola S., Pulvirenti A., Bozzetti F., Montalto F., Ammatuna M., Morabito A., Gennari L. (1996). Liver transplantation for the treatment of small hepatocellular carcinomas in patients with cirrhosis. N. Engl. J. Med..

[4] Yao F.Y., Ferrell L., Bass N.M., Watson J.J., Bacchetti P., Venook A., Ascher N.L., Roberts J.P. (2001). Liver transplantation for hepatocellular carcinoma: Expansion of the tumor size limits does not adversely impact survival. Hepatology.

[5] Yao F.Y., Xiao L., Bass N.M., Kerlan R., Ascher N.L., Roberts J.P. (2007). Liver transplantation for hepatocellular carcinoma: Validation of the UCSF-expanded criteria based on preoperative imaging. Am. J. Transplant..

[6] Toso C., Trotter J., Wei A., Bigam D.L., Shah S., Lancaster J., Grant D.R., Greig P.D., Shapiro A.M., Kneteman N.M. (2008). Total tumor volume predicts risk of recurrence following liver transplantation in patients with hepatocellular carcinoma. Liver Transplant. Off. Publ. Am. Assoc. Study Liver Dis. Int. Liver Transplant. Soc..

[7] Mazzaferro V., Llovet J.M., Miceli R., Bhoori S., Schiavo M., Mariani L., Camerini T., Roayaie S., Schwartz M.E., Grazi G.L. (2009). Predicting survival after liver transplantation in patients with hepatocellular carcinoma beyond the Milan criteria: A retrospective, exploratory analysis. Lancet Oncol..

[8] Sugawara Y., Tamura S., Makuuchi M. (2007). Living donor liver transplantation for hepatocellular carcinoma: Tokyo University series. Dig. Dis..

[9] Lee S.G., Hwang S., Moon D.B., Ahn C.S., Kim K.H., Sung K.B., Ko G.Y., Park K.M., Ha T.Y., Song G.W. (2008). Expanded indication criteria of living donor liver transplantation for hepatocellular carcinoma at one large-volume center. Liver Transplant. Off. Publ. Am. Assoc. Study Liver Dis. Int. Liver Transplant. Soc..

[10] Zheng S.S., Xu X., Wu J., Chen J., Wang W.L., Zhang M., Liang T.B., Wu L.M. (2008). Liver transplantation for hepatocellular carcinoma: Hangzhou experiences. Transplantation.

[11] DuBay D., Sandroussi C., Sandhu L., Cleary S., Guba M., Cattral M.S., McGilvray I., Ghanekar A., Selzner M., Greig P.D. (2011). Liver transplantation for advanced hepatocellular carcinoma using poor tumor differentiation on biopsy as an exclusion criterion. Ann. Surg..

[12] Shirabe K., Itoh S., Yoshizumi T., Soejima Y., Taketomi A., Aishima S., Maehara Y. (2007). The predictors of microvascular invasion in candidates for liver transplantation with hepatocellular carcinoma-with special reference to the serum levels of des-gamma-carboxy prothrombin. J. Surg. Oncol..

[13] Duvoux C., Roudot-Thoraval F., Decaens T., Pessione F., Badran H., Piardi T., Francoz C., Compagnon P., Vanlemmens C., Dumortier J. (2012). Liver transplantation for hepatocellular carcinoma: A model including alpha-fetoprotein improves the performance of Milan criteria. Gastroenterology.

[14] Vivarelli M., Cucchetti A., La Barba G., Ravaioli M., Del Gaudio M., Lauro A., Grazi G.L., Pinna A.D. (2008). Liver transplantation for hepatocellular carcinoma under calcineurin inhibitors: Reassessment of risk factors for tumor recurrence. Ann. Surg..

[15] Mehta N., Dodge J.L., Grab J.D., Yao F.Y. (2020). National Experience on Down-Staging of Hepatocellular Carcinoma Before Liver Transplant: Influence of Tumor Burden, Alpha-Fetoprotein, and Wait Time. Hepatology.

[16] Hameed B., Mehta N., Sapisochin G., Roberts J.P., Yao F.Y. (2014). Alpha-fetoprotein level >1000 ng/mL as an exclusion criterion for liver transplantation in patients with hepatocellular carcinoma meeting the Milan criteria. Liver Transplant. Off. Publ. Am. Assoc. Study Liver Dis. Int. Liver Transplant. Soc..

[17] Toso C., Meeberg G., Hernandez-Alejandro R., Dufour J.F., Marotta P., Majno P., Kneteman N.M. (2015). Total tumor volume and alpha-fetoprotein for selection of transplant candidates with hepatocellular carcinoma: A prospective validation. Hepatology.

[18] Mazzaferro V., Sposito C., Zhou J., Pinna A.D., De Carlis L., Fan J., Cescon M., Di Sandro S., Yi-Feng H., Lauterio A. (2018). Metroticket 2.0 Model for Analysis of Competing Risks of Death After Liver Transplantation for Hepatocellular Carcinoma. Gastroenterology.

[19] Costentin C.E., Bababekov Y.J., Zhu A.X., Yeh H. (2019). Is It Time to Reconsider the Milan Criteria for Selecting Patients With Hepatocellular Carcinoma for Deceased-Donor Liver Transplantation?. Hepatology.

[20] Todo S., Furukawa H., Tada M., Japanese Liver Transplantation Study Group (2007). Extending indication: Role of living donor liver transplantation for hepatocellular carcinoma. Liver Transplant. Off. Publ. Am. Assoc. Study Liver Dis. Int. Liver Transplant. Soc..

[21] Togashi J., Akamastu N., Kokudo N. (2016). Living donor liver transplantation for hepatocellular carcinoma at the University of Tokyo Hospital. Hepatobiliary Surg. Nutr..

[22] Takada Y., Ito T., Ueda M., Sakamoto S., Haga H., Maetani Y., Ogawa K., Ogura Y., Oike F., Egawa H. (2007). Living donor liver transplantation for patients with HCC exceeding the Milan criteria: A proposal of expanded criteria. Dig. Dis..

[23] Shirabe K., Taketomi A., Morita K., Soejima Y., Uchiyama H., Kayashima H., Ninomiya M., Toshima T., Maehara Y. (2011). Comparative evaluation of expanded criteria for patients with hepatocellular carcinoma beyond the Milan criteria undergoing living-related donor liver transplantation. Clin. Transplant..

[24] Kim S.H., Moon D.B., Kim W.J., Kang W.H., Kwon J.H., Jwa E.K., Cho H.D., Ha S.M., Chung Y.K., Lee S.G. (2016). Preoperative prognostic values of alpha-fetoprotein (AFP) and protein induced by vitamin K absence or antagonist-II (PIVKA-II) in patients with hepatocellular carcinoma for living donor liver transplantation. Hepatobiliary Surg. Nutr..

[25] Lee J.H., Cho Y., Kim H.Y., Cho E.J., Lee D.H., Yu S.J., Lee J.W., Yi N.J., Lee K.W., Kim S.H. (2016). Serum Tumor Markers Provide Refined Prognostication in Selecting Liver Transplantation Candidate for Hepatocellular Carcinoma Patients Beyond the Milan Criteria. Ann. Surg..

[26] Shimamura T., Akamatsu N., Fujiyoshi M., Kawaguchi A., Morita S., Kawasaki S., Uemoto S., Kokudo N., Hasegawa K., Ohdan H. (2019). Expanded living-donor liver transplantation criteria for patients with hepatocellular carcinoma based on the Japanese nationwide survey: The 5-5-500 rule—A retrospective study. Transpl. Int..

[27] Halazun K.J., Najjar M., Abdelmessih R.M., Samstein B., Griesemer A.D., Guarrera J.V., Kato T., Verna E.C., Emond J.C., Brown R.S. (2017). Recurrence After Liver Transplantation for Hepatocellular Carcinoma: A New MORAL to the Story. Ann. Surg..

[28] Motomura T., Shirabe K., Mano Y., Muto J., Toshima T., Umemoto Y., Fukuhara T., Uchiyama H., Ikegami T., Yoshizumi T. (2013). Neutrophil-lymphocyte ratio reflects hepatocellular carcinoma recurrence after liver transplantation via inflammatory microenvironment. J. Hepatol..

[29] Mehta N., Dodge J.L., Roberts J.P., Yao F.Y. (2018). Validation of the prognostic power of the RETREAT score for hepatocellular carcinoma recurrence using the UNOS database. Am. J. Transplant..

[30] Sasaki K., Firl D.J., Hashimoto K., Fujiki M., Diago-Uso T., Quintini C., Eghtesad B., Fung J.J., Aucejo F.N., Miller C.M. (2017). Development and validation of the HALT-HCC score to predict mortality in liver transplant recipients with hepatocellular carcinoma: A retrospective cohort analysis. Lancet Gastroenterol. Hepatol..

[31] Sasaki K., Morioka D., Conci S., Margonis G.A., Sawada Y., Ruzzenente A., Kumamoto T., Iacono C., Andreatos N., Guglielmi A. (2018). The Tumor Burden Score: A New “Metro-ticket” Prognostic Tool For Colorectal Liver Metastases Based on Tumor Size and Number of Tumors. Ann. Surg..

[32] Firl D.J., Sasaki K., Agopian V.G., Gorgen A., Kimura S., Dumronggittigule W., McVey J.C., Iesari S., Mennini G., Vitale A. (2020). Charting the Path Forward for Risk Prediction in Liver Transplant for Hepatocellular Carcinoma: International Validation of HALTHCC Among 4089 Patients. Hepatology.

[33] Roayaie S., Schwartz J.D., Sung M.W., Emre S.H., Miller C.M., Gondolesi G.E., Krieger N.R., Schwartz M.E. (2004). Recurrence of hepatocellular carcinoma after liver transplant: Patterns and prognosis. Liver Transplant. Off. Publ. Am. Assoc. Study Liver Dis. Int. Liver Transplant. Soc..

[34] Pawlik T.M., Gleisner A.L., Anders R.A., Assumpcao L., Maley W., Choti M.A. (2007). Preoperative assessment of hepatocellular carcinoma tumor grade using needle biopsy: Implications for transplant eligibility. Ann. Surg..

[35] Rodriguez-Peralvarez M., Luong T.V., Andreana L., Meyer T., Dhillon A.P., Burroughs A.K. (2013). A systematic review of microvascular invasion in hepatocellular carcinoma: Diagnostic and prognostic variability. Ann. Surg. Oncol..

[36] Liu C., Xiao G.Q., Yan L.N., Li B., Jiang L., Wen T.F., Wang W.T., Xu M.Q., Yang J.Y. (2013). Value of alpha-fetoprotein in association with clinicopathological features of hepatocellular carcinoma. World J. Gastroenterol..

[37] Hakeem A.R., Young R.S., Marangoni G., Lodge J.P., Prasad K.R. (2012). Systematic review: The prognostic role of alpha-fetoprotein following liver transplantation for hepatocellular carcinoma. Aliment. Pharmacol. Ther..

[38] She W.H., Chan A.C.Y., Cheung T.T., Lo C.M., Chok K.S.H. (2018). Survival outcomes of liver transplantation for hepatocellular carcinoma in patients with normal, high and very high preoperative alpha-fetoprotein levels. World J. Hepatol..

[39] Mehta N., Bhangui P., Yao F.Y., Mazzaferro V., Toso C., Akamatsu N., Durand F., Ijzermans J., Polak W., Zheng S. (2020). Liver Transplantation for Hepatocellular Carcinoma. Working Group Report from the ILTS Transplant Oncology Consensus Conference. Transplantation.

[40] Lu Y., Zhu M., Li W., Lin B., Dong X., Chen Y., Xie X., Guo J., Li M. (2016). Alpha fetoprotein plays a critical role in promoting metastasis of hepatocellular carcinoma cells. J. Cell Mol. Med..

[41] Wang X., Wang Q. (2018). Alpha-Fetoprotein and Hepatocellular Carcinoma Immunity. Can. J. Gastroenterol. Hepatol..

[42] Gao F.J., Cui S.X., Chen M.H., Cheng Y.N., Sun L.R., Ward S.G., Kokudo N., Tang W., Qu X.J. (2008). Des-gamma-carboxy prothrombin increases the expression of angiogenic factors in human hepatocellular carcinoma cells. Life Sci..

[43] Wang S.B., Cheng Y.N., Cui S.X., Zhong J.L., Ward S.G., Sun L.R., Chen M.H., Kokudo N., Tang W., Qu X.J. (2009). Des-gamma-carboxy prothrombin stimulates human vascular endothelial cell growth and migration. Clin. Exp. Metastasis.

[44] Pote N., Cauchy F., Albuquerque M., Voitot H., Belghiti J., Castera L., Puy H., Bedossa P., Paradis V. (2015). Performance of PIVKA-II for early hepatocellular carcinoma diagnosis and prediction of microvascular invasion. J. Hepatol..

[45] Okuda H., Nakanishi T., Takatsu K., Saito A., Hayashi N., Yamamoto M., Takasaki K., Nakano M. (2001). Comparison of clinicopathological features of patients with hepatocellular carcinoma seropositive for alpha-fetoprotein alone and those seropositive for des-gamma-carboxy prothrombin alone. J. Gastroenterol. Hepatol..

[46] Hong Y.M., Cho M., Yoon K.T., Chu C.W., Yang K.H., Park Y.M., Rhu J.H. (2017). Risk factors of early recurrence after curative hepatectomy in hepatocellular carcinoma. Tumour Biol..

[47] Chaiteerakij R., Zhang X., Addissie B.D., Mohamed E.A., Harmsen W.S., Theobald P.J., Peters B.E., Balsanek J.G., Ward M.M., Giama N.H. (2015). Combinations of biomarkers and Milan criteria for predicting hepatocellular carcinoma recurrence after liver transplantation. Liver Transplant. Off. Publ. Am. Assoc. Study Liver Dis. Int. Liver Transplant. Soc..

[48] Halazun K.J., Hardy M.A., Rana A.A., Woodland D.C.t., Luyten E.J., Mahadev S., Witkowski P., Siegel A.B., Brown R.S., Emond J.C. (2009). Negative impact of neutrophil-lymphocyte ratio on outcome after liver transplantation for hepatocellular carcinoma. Ann. Surg..

[49] Xu Z.G., Ye C.J., Liu L.X., Wu G., Zhao Z.X., Wang Y.Z., Shi B.Q., Wang Y.H. (2018). The pretransplant neutrophil-lymphocyte ratio as a new prognostic predictor after liver transplantation for hepatocellular cancer: A systematic review and meta-analysis. Biomark Med..

[50] Yoshizumi T., Ikegami T., Yoshiya S., Motomura T., Mano Y., Muto J., Ikeda T., Soejima Y., Shirabe K., Maehara Y. (2013). Impact of tumor size, number of tumors and neutrophil-to-lymphocyte ratio in liver transplantation for recurrent hepatocellular carcinoma. Hepatol. Res..

[51] Na G.H., Kim D.G., Han J.H., Kim E.Y., Lee S.H., Hong T.H., You Y.K. (2014). Inflammatory markers as selection criteria of hepatocellular carcinoma in living-donor liver transplantation. World J. Gastroenterol..

[52] Shindoh J., Sugawara Y., Nagata R., Kaneko J., Tamura S., Aoki T., Sakamoto Y., Hasegawa K., Tanaka T., Kokudo N. (2014). Evaluation methods for pretransplant oncologic markers and their prognostic impacts in patient undergoing living donor liver transplantation for hepatocellular carcinoma. Transpl. Int..

[53] Lai Q., Castro Santa E., Rico Juri J.M., Pinheiro R.S., Lerut J. (2014). Neutrophil and platelet-to-lymphocyte ratio as new predictors of dropout and recurrence after liver transplantation for hepatocellular cancer. Transpl. Int..

[54] Harimoto N., Yoshizumi T., Shimagaki T., Nagatsu A., Motomura T., Harada N., Okabe H., Itoh S., Ikegami T., Uchiyama H. (2016). Inflammation-based Prognostic Score in Patients with Living Donor Liver Transplantation for Hepatocellular Carcinoma. Anticancer Res..

[55] Wang W., Ye Y., Wang T., Zhang F., Geng L., Yu J., Zhou L., Yan S., Zheng S. (2016). Prognostic prediction of male recipients selected for liver transplantation: With special attention to neutrophil to lymphocyte ratio. Hepatol. Res..

[56] Kusumanto Y.H., Dam W.A., Hospers G.A., Meijer C., Mulder N.H. (2003). Platelets and granulocytes, in particular the neutrophils, form important compartments for circulating vascular endothelial growth factor. Angiogenesis.

[57] Bambace N.M., Holmes C.E. (2011). The platelet contribution to cancer progression. J. Thromb. Haemost..

[58] Sweeney M.J., Ashmore J., Morris H.P., Weber G. (1963). Comparative Biochemistry Hepatomas. Iv. Isotope Studies of Glucose and Fructose Metabolism in Liver Tumors of Different Growth Rates. Cancer Res..

[59] Torizuka T., Tamaki N., Inokuma T., Magata Y., Sasayama S., Yonekura Y., Tanaka A., Yamaoka Y., Yamamoto K., Konishi J. (1995). In vivo assessment of glucose metabolism in hepatocellular carcinoma with FDG-PET. J. Nucl. Med..

[60] Lin C.C., Chen C.L. (2016). Living donor liver transplantation for hepatocellular carcinoma achieves better outcomes. Hepatobiliary Surg. Nutr..

[61] Seo S., Hatano E., Higashi T., Hara T., Tada M., Tamaki N., Iwaisako K., Ikai I., Uemoto S. (2007). Fluorine-18 fluorodeoxyglucose positron emission tomography predicts tumor differentiation, P-glycoprotein expression, and outcome after resection in hepatocellular carcinoma. Clin. Cancer Res..

[62] Lee S.D., Kim S.H., Kim S.K., Kim Y.K., Park S.J. (2015). Clinical Impact of 18F-Fluorodeoxyglucose Positron Emission Tomography/Computed Tomography in Living Donor Liver Transplantation for Advanced Hepatocellular Carcinoma. Transplantation.

[63] Lee S.D., Kim S.H., Kim Y.K., Kim C., Kim S.K., Han S.S., Park S.J. (2013). (18)F-FDG-PET/CT predicts early tumor recurrence in living donor liver transplantation for hepatocellular carcinoma. Transpl. Int..

[64] Kornberg A., Kupper B., Tannapfel A., Buchler P., Krause B., Witt U., Gottschild D., Friess H. (2012). Patients with non-[18 F]fludeoxyglucose-avid advanced hepatocellular carcinoma on clinical staging may achieve long-term recurrence-free survival after liver transplantation. Liver Transpl..

[65] Kornberg A., Freesmeyer M., Barthel E., Jandt K., Katenkamp K., Steenbeck J., Sappler A., Habrecht O., Gottschild D., Settmacher U. (2009). 18F-FDG-uptake of hepatocellular carcinoma on PET predicts microvascular tumor invasion in liver transplant patients. Am. J. Transplant..

[66] Yang S.H., Suh K.S., Lee H.W., Cho E.H., Cho J.Y., Cho Y.B., Yi N.J., Lee K.U. (2006). The role of (18)F-FDG-PET imaging for the selection of liver transplantation candidates among hepatocellular carcinoma patients. Liver Transpl..

[67] Bailly M., Venel Y., Orain I., Salame E., Ribeiro M.J. (2016). 18F-FDG PET in Liver Transplantation Setting of Hepatocellular Carcinoma: Predicting Histology?. Clin. Nucl. Med..

[68] Kornberg A., Kupper B., Thrum K., Katenkamp K., Steenbeck J., Sappler A., Habrecht O., Gottschild D. (2009). Increased 18F-FDG uptake of hepatocellular carcinoma on positron emission tomography independently predicts tumor recurrence in liver transplant patients. Transplant. Proc..

[69] Lee J.W., Paeng J.C., Kang K.W., Kwon H.W., Suh K.S., Chung J.K., Lee M.C., Lee D.S. (2009). Prediction of tumor recurrence by 18F-FDG PET in liver transplantation for hepatocellular carcinoma. J. Nucl. Med..

[70] Hsu C.C., Chen C.L., Wang C.C., Lin C.C., Yong C.C., Wang S.H., Liu Y.W., Lin T.L., Lee W.F., Lin Y.H. (2016). Combination of FDG-PET and UCSF Criteria for Predicting HCC Recurrence After Living Donor Liver Transplantation. Transplantation.

[71] Kornberg A., Witt U., Schernhammer M., Kornberg J., Ceyhan G.O., Mueller K., Friess H., Thrum K. (2017). Combining (18)F-FDG positron emission tomography with Up-to-seven criteria for selecting suitable liver transplant patients with advanced hepatocellular carcinoma. Sci. Rep..

[72] Takada Y., Kaido T., Shirabe K., Nagano H., Egawa H., Sugawara Y., Taketomi A., Takahara T., Wakabayashi G., Nakanishi C. (2017). Significance of preoperative fluorodeoxyglucose-positron emission tomography in prediction of tumor recurrence after liver transplantation for hepatocellular carcinoma patients: A Japanese multicenter study. J. Hepatobiliary Pancreat. Sci..

[73] Hong G., Suh K.S., Suh S.W., Yoo T., Kim H., Park M.S., Choi Y., Paeng J.C., Yi N.J., Lee K.W. (2016). Alpha-fetoprotein and (18)F-FDG positron emission tomography predict tumor recurrence better than Milan criteria in living donor liver transplantation. J. Hepatol..

[74] Lin C.Y., Liao C.W., Chu L.Y., Yen K.Y., Jeng L.B., Hsu C.N., Lin C.L., Kao C.H. (2017). Predictive Value of 18F-FDG PET/CT for Vascular Invasion in Patients With Hepatocellular Carcinoma Before Liver Transplantation. Clin. Nucl. Med..

[75] Ahn S.Y., Lee J.M., Joo I., Lee E.S., Lee S.J., Cheon G.J., Han J.K., Choi B.I. (2015). Prediction of microvascular invasion of hepatocellular carcinoma using gadoxetic acid-enhanced MR and (18)F-FDG PET/CT. Abdom. Imaging.

[76] Li J., Han X., Yu X., Xu Z., Yang G., Liu B., Xiu P. (2018). Clinical applications of liquid biopsy as prognostic and predictive biomarkers in hepatocellular carcinoma: Circulating tumor cells and circulating tumor DNA. J. Exp. Clin. Cancer Res..

[77] Wang S., Zheng Y., Liu J., Huo F., Zhou J. (2018). Analysis of circulating tumor cells in patients with hepatocellular carcinoma recurrence following liver transplantation. J. Investig Med..

[78] Xue F., Shi S., Zhang Z., Xu C., Zheng J., Qin T., Qian Z., Zhao X., Tong Y., Xia L. (2018). Application of a novel liquid biopsy in patients with hepatocellular carcinoma undergoing liver transplantation. Oncol. Lett..

[79] Wang P.X., Xu Y., Sun Y.F., Cheng J.W., Zhou K.Q., Wu S.Y., Hu B., Zhang Z.F., Guo W., Cao Y. (2021). Detection of circulating tumour cells enables early recurrence prediction in hepatocellular carcinoma patients undergoing liver transplantation. Liver Int. Off. J. Int. Assoc. Study Liver.

[80] Marubashi S., Dono K., Nagano H., Sugita Y., Asaoka T., Hama N., Miyamoto A., Takeda Y., Umeshita K., Monden M. (2007). Detection of AFP mRNA-expressing cells in the peripheral blood for prediction of HCC recurrence after living donor liver transplantation. Transpl. Int..

[81] Otto G., Herber S., Heise M., Lohse A.W., Monch C., Bittinger F., Hoppe-Lotichius M., Schuchmann M., Victor A., Pitton M. (2006). Response to transarterial chemoembolization as a biological selection criterion for liver transplantation in hepatocellular carcinoma. Liver Transplant. Off. Publ. Am. Assoc. Study Liver Dis. Int. Liver Transplant. Soc..

[82] Bargellini I., Vignali C., Cioni R., Petruzzi P., Cicorelli A., Campani D., De Simone P., Filipponi F., Bartolozzi C. (2010). Hepatocellular carcinoma: CT for tumor response after transarterial chemoembolization in patients exceeding Milan criteria--selection parameter for liver transplantation. Radiology.

[83] Cucchetti A., Serenari M., Sposito C., Di Sandro S., Mosconi C., Vicentin I., Garanzini E., Mazzaferro V., De Carlis L., Golfieri R. (2020). Including mRECIST in the Metroticket 2.0 criteria improves prediction of hepatocellular carcinoma-related death after liver transplant. J. Hepatol..

[84] Mehta N., Dodge J.L., Goel A., Roberts J.P., Hirose R., Yao F.Y. (2013). Identification of liver transplant candidates with hepatocellular carcinoma and a very low dropout risk: Implications for the current organ allocation policy. Liver Transplant. Off. Publ. Am. Assoc. Study Liver Dis. Int. Liver Transplant. Soc..

[85] Agopian V.G., Harlander-Locke M.P., Ruiz R.M., Klintmalm G.B., Senguttuvan S., Florman S.S., Haydel B., Hoteit M., Levine M.H., Lee D.D. (2017). Impact of Pretransplant Bridging Locoregional Therapy for Patients With Hepatocellular Carcinoma Within Milan Criteria Undergoing Liver Transplantation: Analysis of 3601 Patients From the US Multicenter HCC Transplant Consortium. Ann. Surg..

[86] Kim D.J., Clark P.J., Heimbach J., Rosen C., Sanchez W., Watt K., Charlton M.R. (2014). Recurrence of Hepatocellular Carcinoma: Importance of mRECIST Response to Chemoembolization and Tumor Size. Am. J. Transplant..

[87] DiNorcia J., Florman S.S., Haydel B., Tabrizian P., Ruiz R.M., Klintmalm G.B., Senguttuvan S., Lee D.D., Taner C.B., Verna E.C. (2020). Pathologic Response to Pretransplant Locoregional Therapy is Predictive of Patient Outcome After Liver Transplantation for Hepatocellular Carcinoma: Analysis From the US Multicenter HCC Transplant Consortium. Ann. Surg..

[88] Giard J.M., Mehta N., Dodge J.L., Roberts J.P., Yao F.Y. (2018). Alpha-Fetoprotein Slope >7.5 ng/mL per Month Predicts Microvascular Invasion and Tumor Recurrence After Liver Transplantation for Hepatocellular Carcinoma. Transplantation.

[89] Ogawa K., Kaido T., Okajima H., Fujimoto Y., Yoshizawa A., Yagi S., Hori T., Iida T., Takada Y., Uemoto S. (2019). Impact of pretreatments on outcomes after living donor liver transplantation for hepatocellular carcinoma. J. Hepatobiliary Pancreat. Sci..

[90] Toniutto P., Fumolo E., Fornasiere E., Bitetto D. (2021). Liver Transplantation in Patients with Hepatocellular Carcinoma beyond the Milan Criteria: A Comprehensive Review. J. Clin. Med..

[91] Lai Q., Nicolini D., Inostroza Nunez M., Iesari S., Goffette P., Agostini A., Giovagnoni A., Vivarelli M., Lerut J. (2016). A Novel Prognostic Index in Patients With Hepatocellular Cancer Waiting for Liver Transplantation: Time-Radiological-response-Alpha-fetoprotein-INflammation (TRAIN) Score. Ann. Surg..

[92] Firl D.J., Kimura S., McVey J., Hashimoto K., Yeh H., Miller C.M., Markmann J.F., Sasaki K., Aucejo F.N. (2018). Reframing the approach to patients with hepatocellular carcinoma: Longitudinal assessment with hazard associated with liver transplantation for HCC (HALTHCC) improves ablate and wait strategy. Hepatology.

[93] Asrani S.K., Devarbhavi H., Eaton J., Kamath P.S. (2019). Burden of liver diseases in the world. J. Hepatol..

[94] Schaubel D.E., Guidinger M.K., Biggins S.W., Kalbfleisch J.D., Pomfret E.A., Sharma P., Merion R.M. (2009). Survival benefit-based deceased-donor liver allocation. Am. J. Transplant..

[95] Delmonico F.L., Jenkins R.L., Freeman R., Vacanti J., Bradley J., Dienstag J.L., Trey C., Lewis W.D., Lillehei C.W., Auchincloss H. (1992). The high-risk liver allograft recipient. Should allocation policy consider outcome?. Arch. Surg..

[96] Halldorson J.B., Bakthavatsalam R., Fix O., Reyes J.D., Perkins J.D. (2009). D-MELD, a simple predictor of post liver transplant mortality for optimization of donor/recipient matching. Am. J. Transplant..

[97] Rana A., Hardy M.A., Halazun K.J., Woodland D.C., Ratner L.E., Samstein B., Guarrera J.V., Brown R.S., Emond J.C. (2008). Survival outcomes following liver transplantation (SOFT) score: A novel method to predict patient survival following liver transplantation. Am. J. Transplant..

[98] Dutkowski P., Oberkofler C.E., Slankamenac K., Puhan M.A., Schadde E., Müllhaupt B., Geier A., Clavien P.A. (2011). Are there better guidelines for allocation in liver transplantation? A novel score targeting justice and utility in the model for end-stage liver disease era. Ann. Surg..

[99] Merion R.M., Schaubel D.E., Dykstra D.M., Freeman R.B., Port F.K., Wolfe R.A. (2005). The survival benefit of liver transplantation. Am. J. Transplant..

[100] Vitale A., Volk M.L., De Feo T.M., Burra P., Frigo A.C., Ramirez Morales R., De Carlis L., Belli L., Colledan M., Fagiuoli S. (2014). A method for establishing allocation equity among patients with and without hepatocellular carcinoma on a common liver transplant waiting list. J. Hepatol..

[101] OPTN Policy Policy 9: Allocation of Livers and Liver-Intestines. https://optn.traansplant.hrsa.gov/.

[102] Brahmania M., Marquez V., Kneteman N.M., Bhat M., Marleau D., Wong P., Peletekian K.M., Burak K.W., Congly S.E. (2019). Canadian liver transplant allocation for hepatocellular carcinoma. J. Hepatol..

[103] NHS Policies and Guidance Liver Selection and Allocation. Liver Selection Policy—POL 195. https://nhsbtdbe.blob.core.windows.net/.

[104] Eurotransplant Manual—Chapter 5. ET Liver Allocation System. https://www.eurotransplant.org.

[105] Rodríguez-Perálvarez M., Gómez-Bravo M., Sánchez-Antolín G., De la Rosa G., Bilbao I., Colmenero J., on behalf of the Spanish Society of Liver Transplantation (SETH) Consensus Panel (2021). Expanding Indications of Liver Transplantation in Spain: Consensus Statement and Recommendations by the Spanish Society of Liver Transplantation. Transplantation.

[106] Durand F., Antoine C., Soubrane O. (2019). Liver Transplantation in France. Liver Transpl..

[107] Cillo U., Burra P., Mazzaferro V., Belli L., Pinna A.D., Spada M., Nanni Costa A., Toniutto P., I-BELT (Italian Board of Experts in the Field of Liver Transplantation) (2015). A Multistep, Consensus-Based Approach to Organ Allocation in Liver Transplantation: Toward a “Blended Principle Model”. Am. J. Transplant..

[108] Lee J., Lee J.G., Jung I., Joo D.J., Kim S.I., Kim M.S., Allocation A.C.o.I.L. (2019). Development of a Korean Liver Allocation System using Model for End Stage Liver Disease Scores: A Nationwide, Multicenter study. Sci. Rep..

[109] Yao F.Y., Mehta N., Flemming J., Dodge J., Hameed B., Fix O., Hirose R., Fidelman N., Kerlan R.K., Roberts J.P. (2015). Downstaging of hepatocellular cancer before liver transplant: Long-term outcome compared to tumors within Milan criteria. Hepatology.

[110] Ravaioli M., Grazi G.L., Piscaglia F., Trevisani F., Cescon M., Ercolani G., Vivarelli M., Golfieri R., D’Errico Grigioni A., Panzini I. (2008). Liver transplantation for hepatocellular carcinoma: Results of down-staging in patients initially outside the Milan selection criteria. Am. J. Transplant..

[111] Lee S., Kim K.W., Song G.W., Kwon J.H., Hwang S., Kim K.H., Ahn C.S., Moon D.B., Park G.C., Lee S.G. (2020). The Real Impact of Bridging or Downstaging on Survival Outcomes after Liver Transplantation for Hepatocellular Carcinoma. Liver Cancer.

[112] Mehta N., Guy J., Frenette C.T., Dodge J.L., Osorio R.W., Minteer W.B., Roberts J.P., Yao F.Y. (2018). Excellent Outcomes of Liver Transplantation Following Down-Staging of Hepatocellular Carcinoma to Within Milan Criteria: A Multicenter Study. Clin. Gastroenterol. Hepatol..

[113] Kardashian A., Florman S.S., Haydel B., Ruiz R.M., Klintmalm G.B., Lee D.D., Taner C.B., Aucejo F., Tevar A.D., Humar A. (2020). Liver Transplantation Outcomes in a U.S. Multicenter Cohort of 789 Patients With Hepatocellular Carcinoma Presenting Beyond Milan Criteria. Hepatology.

[114] Yao F.Y., Fidelman N. (2016). Reassessing the boundaries of liver transplantation for hepatocellular carcinoma: Where do we stand with tumor down-staging?. Hepatology.

[115] Mehta N., Frenette C., Tabrizian P., Hoteit M., Guy J., Parikh N., Ghaziani T.T., Dhanasekaran R., Dodge J.L., Natarajan B. (2021). Downstaging Outcomes for Hepatocellular Carcinoma: Results From the Multicenter Evaluation of Reduction in Tumor Size before Liver Transplantation (MERITS-LT) Consortium. Gastroenterology.

[116] Chapman W.C., Garcia-Aroz S., Vachharajani N., Fowler K., Saad N., Lin Y., Wellen J., Tan B., Khan A.S., Doyle M.B. (2017). Liver Transplantation for Advanced Hepatocellular Carcinoma after Downstaging Without Up-Front Stage Restrictions. J. Am. Coll. Surg..

[117] Clavien P.-A., Lesurtel M., Bossuyt P.M.M., Gores G.J., Langer B., Perrier A. (2012). Recommendations for liver transplantation for hepatocellular carcinoma: An international consensus conference report. Lancet Oncol..

[118] European Association for the Study of the Liver (2018). EASL Clinical Practice Guidelines: Management of hepatocellular carcinoma. J. Hepatol..

[119] Heimbach J.K., Kulik L.M., Finn R.S., Sirlin C.B., Abecassis M.M., Roberts L.R., Zhu A.X., Murad M.H., Marrero J.A. (2018). AASLD guidelines for the treatment of hepatocellular carcinoma. Hepatology.

[120] Mazzaferro V., Citterio D., Bhoori S., Bongini M., Miceli R., De Carlis L., Colledan M., Salizzoni M., Romagnoli R., Antonelli B. (2020). Liver transplantation in hepatocellular carcinoma after tumour downstaging (XXL): A randomised, controlled, phase 2b/3 trial. Lancet Oncol..

[121] Tabrizian P., Florman S.S., Schwartz M.E. (2021). PD-1 inhibitor as bridge therapy to liver transplantation?. Am. J. Transplant..

